# Tape-Worm in Denmark

**Published:** 1887-12

**Authors:** H. Krabbe


### *TAPE-WORM IN DENMARK.*

In 1880 I reported 200 cases of cestodes occurring in man, and since that date I have observed a new series of 100 cases.

Of these 300 cases more than half were treated in Copenhagen, and of the last 100, eighty-one were observed in this city.

According to the sex of the patients the different varieties of tapeworm were distributed as follows:

| Sex. | T. saginata. | T. solium. | T. cucumerina. | Both. latus. |
|---|---|---|---|---|
| Males . . . . . | 59 | 17 | 6 | 3 |
| Females . . . . | 124 | 57 | 2 | 22 |

According to the age of the patient the varieties of the worm were distributed as follows:

| Age. | T. saginata. | T. solium. | T. cucumerina. | Both. latus. |
|---|---|---|---|---|
| Under 1 year . . | . | . | 9 | . |
| 1 to 10 years . . | 11 | 7 | . | . |
| 10 to 20 years . . | 14 | 5 | . | . |
| 20 to 30 years . . | 79 | 15 | . | 10 |
| 30 to 40 years . . | 35 | 15 | . | 6 |
| 40 to 50 years . . | 20 | 4 | . | 5 |
| 50 to 60 years . . | 15 | 2 | . | . |
| 60 to 70 years . . | 4 | . | . | 2 |

Tænia solium has been diminishing in frequency, especially since 1880, while tænia saginata has perhaps increased.

| | T. saginata. | T. solium. | T. cucumerina. | Both. latus. |
|---|---|---|---|---|
| Case before 1869 . . | 37 | 53 | 1 | 9 |
| From 1860 to 1880 . | 67 | 19 | 4 | 11 |
| From 1880 to 1887 . | 86 | 5 | 4 | 5 |

With regard to the number of worms in a single patient the cases were distributed as follows:

T. saginata . . 190 cases; always single.
T. solium . . . 77 cases; 13 cases with more than one worm.
T. cucumerina. 9 cases; 3 cases with two worms.
Bots. latus . . 25 cases; 5 cases with from 2 to 14 worms.

The remedy employed most frequently was oleo-resin of male fern, ʒiiss., followed by a laxative of calomel and jalap, āā gr. viii.—*H. Krabbe in Nordiskt Medicinskt Arkiv.*

